# Trace Element Deficiency in Axial Spondyloarthritis and Psoriatic Arthritis in Relation to Markers of Inflammation and Remission

**DOI:** 10.3390/ijms26104924

**Published:** 2025-05-21

**Authors:** Uwe Gröber, Styliani Tsiami, Thilo Samson Chillon, Evangelos Rousis, Klaus Kisters, Sophia Karmeli, Uta Kiltz, Lutz Schomburg, Xenofon Baraliakos

**Affiliations:** 1 Institute for Experimental Endocrinology, Charité-Universitätsmedizin Berlin, 10117 Berlin, Germany; ug@vitaminspur.de (U.G.); thilo.chillon@charite.de (T.S.C.); sophia.karmeli@charite.de (S.K.); 2 Rheumazentrum Ruhrgebiet, 44649 Herne, Germany; styliani.tsiami@elisabethgruppe.de (S.T.); evangelos.rousis@elisabethgruppe.de (E.R.); uta.kiltz@elisabethgruppe.de (U.K.); 3 Medical Faculty, Ruhr-Universität Bochum, 44649 Herne, Germany; 4 Med Univ Poliklinik, 48149 Münster, Germany; k.kisters@dialyse-herne.de

**Keywords:** copper, selenium, selenoprotein, zinc, inflammatory rheumatic musculoskeletal diseases, C-reactive protein, supplementation

## Abstract

Selenium (Se) is an essential micronutrient for antioxidant defense. Selenoproteins are involved in metabolic and signaling pathways of autoimmune and autoinflammatory diseases. Copper (Cu) and zinc (Zn) are integral components of key enzymes and regulatory proteins. Trace element (TE) dysregulations have potential relevance as disease biomarkers. In this study, we compare TE status and TE profiles between patients with axial spondyloarthritis (axSpA) and psoriatic arthritis (PsA), and relate the results to markers of inflammation, remission, and healthy controls. Serum TE was measured using total reflection X-ray fluorescence. The Se transporter SELENOP and extracellular glutathione peroxidase (GPx3) were determined by ELISA and enzymatic assay, respectively. Both groups of patients (axSpA; n = 84, and PsA; n = 76) displayed TE deficiency compared to healthy European adults. Serum Cu, Se, Zn, SELENOP, and GPx3 levels were not different between the groups. The serum Cu and Cu-Zn ratio correlated positively with C-reactive protein (CRP) and erythrocyte sedimentation rate (ESR). An inverse correlation of serum Se, Zn, and SELENOP with CRP was observed in axSpA, but not in PsA. On average, all TE, including the inflammation-responsive Cu levels, were below reference ranges, indicating a TE deficiency in both groups. Increasing CRP was associated with low SELENOP levels, suggesting personalized Se substitution as indicated to maintain systemic Se supply and protect from ferroptotic cell loss under inflammatory conditions.

## 1. Introduction

The term spondyloarthritis (SpA) encompasses a group of inflammatory rheumatic diseases with some clinical and genetic similarities, particularly characterized by inflammation of the axial skeleton, peripheral joints, and tendon insertions, and association with the major histocompatibility complex (MHC) class I human leukocyte antigen (HLA)-B27 [1]. SpA includes both axial (axSpA) and peripheral forms of the disease, with the main representative of the latter being psoriatic arthritis (PsA). Axial SpA (axSpA) is a chronic inflammatory rheumatic disease with onset in early adulthood. The disease is characterized by acute or chronic inflammation in the area of the sacroiliac joints (SIJ, sacroiliitis) and affection of structures of the axial skeleton, leading to findings such as spondylitis, spondylodiscitis, and facet joint arthritis as well as arthritis/enthesitis of the costovertebral and costosternal joints. Axial spondyloarthritis includes the stage of non-radiographic axSpA (nr-axSpA), which by definition has no definite structural radiographic changes, and the stage of radiographic axial spondyloarthritis (r-axSpA, formerly known as ankylosing spondylitis, AS), which is characterized by structural bone changes in SIJ on conventional X- and [2]. Psoriatic arthritis (PsA) is a frequent progressive, erosive, chronic systemic inflammatory disease that can manifest itself with six different clinical domains of affection, including peripheral arthritis, dactylitis, enthesitis, psoriasis, psoriatic nail disease, and axial disease [3].

Previous studies have assessed different biomarkers of micronutrients in relation to inflammatory rheumatic musculoskeletal diseases (iRMD). Among the most intensively studied topics is the vitamin D status, and its deficiency is described as a risk factor for disease susceptibility and severe course [4]. From the group of minerals, the essential trace elements copper (Cu), selenium (Se), and zinc (Zn) have been investigated. Selenium is an essential micronutrient that influences various aspects of human health, including optimal immune response. Through its incorporation into selenoproteins, Se is involved in the regulation of oxidative stress, redox status, and other important cellular processes in almost all tissues and cell types, including those involved in innate and adaptive immune responses [5]. Selenoproteins are involved in reproduction, thyroid activity, DNA synthesis, muscle function, and various other metabolic pathways. Cellular glutathione peroxidase (GPx1) was the first mammalian enzyme to be characterized as a selenoprotein [6]. The identification of the iodothyronine deiodinases as selenoproteins linked the trace element with the endocrine system and thyroid hormone metabolism [7]. A strong interaction and general dependence of autoimmune and autoinflammatory diseases with the Se status and the expression of selenoproteins has been described in a variety of diseases, in particular in autoimmune thyroid disease, systemic lupus erythematosus, and rheumatic diseases [8,9,10]. The lipid oxidation-induced cell death pathway of ferroptosis has been shown to depend on the Se status and expression of the selenoprotein GPx4 and contributes in particular to autoimmune disease risks and damage from ongoing inflammation [11,12]. Besides Se, the trace elements Cu and Zn are also of importance for the immune system and have been described as potential biomarkers reflecting inflammatory cytokine status and lymphocyte activity in rheumatic diseases [13,14,15].

In general, the previously published results indicate a correlation of micronutrient dysregulation with inflammatory activity, as well as marked differences in relation to the population studied, disease duration, treatment modalities, and analytical matrix chosen [16].

In light of this knowledge, we prospectively analyzed the above-mentioned TE in axSpA and PsA in order to identify similarities and differences in the TE status and TE profiles between the two diseases.

## 2. Results

### 2.1. Characterization of the Study Cohort

The observational study recruited n = 84 patients with axSpA and n = 76 with PsA, with a median age of 43.0 and 52.5 years, respectively, with a relatively high number of subjects for whom weight was not available, and with 30–38% of subjects positive for CRP (Table 1). No control subjects were recruited for this particular study, as a solid database for the trace elements and their biomarkers was available from a previous large observational cross-sectional analysis of healthy adult subjects, which had been analyzed by the same methods and technology [17].

### 2.2. Biomarkers of Selenium Status

**Figure 1:**
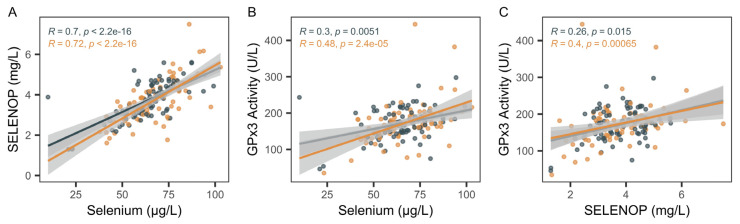
Analysis of biomarkers of Se status in patients with PsA or axSpA. All samples were analyzed for total serum Se, GPx3 activity, and SELENOP concentration. (**A**) Linear positive correlations between Se and SELENOP were observed in both groups of patients, with a relatively steep slope of the interaction. (**B**) Both groups of patients displayed a linear correlation between serum Se and GPx3 activity and (**C**) between serum SELENOP and GPx3 activity. Analysis by non-parametric Spearman correlation. R and *p*-values are indicated. Data for axSpA are indicated in black; data for PsA are indicated in orange.

The full set of serum samples from the patients with a diagnosis of axSpA or PsA was analyzed for TE concentrations and two protein biomarkers of Se status, namely GPx3 activity and SELENOP levels. Consistent positive linear associations of the three Se status biomarkers were observed (Figure 1), with a particularly strong correlation between total Se and SELENOP concentrations (Figure 1A). GPx3 and Se, as well as GPx3 and SELENOP, showed a steeper and more significant correlation in the patients with PsA as compared to the patients with axSpA (Figure 1B,C).

### 2.3. Comparison of Trace Element Status in axSpA Versus PsA

**Figure 2:**
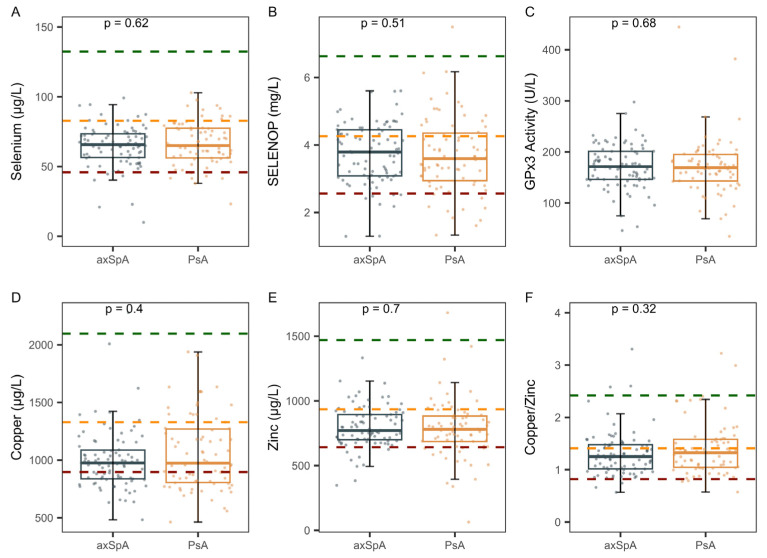
Comparison of biomarkers of trace element status between the groups of patients with ankylosing spondylitis (axSpA) and psoriatic arthritis (PsA). All samples were analyzed for three biomarkers of Se status, as well as for serum Cu, Zn, and their ratio. The direct comparison showed no significant differences between the two groups in (**A**) serum Se, (**B**) SELENOP, (**C**) GPx3 activity, (**D**) total Cu, (**E**) Zn, or (**F**) the Cu-Zn ratio. On average, both groups displayed a relative deficiency in the TE parameters compared to healthy European adults. The green dashed lines indicate the 97.5th percentiles, the orange lines denote the medians (not available for GPx3), and the red dashed lines indicate the 2.5th percentiles of the cross-sectional EPIC study. Analysis by nonparametric group comparisons. *p*-values are indicated.

Next, the two groups of patients were compared with respect to the biomarkers of Se status and the trace elements Cu and Zn, as well as the Cu-Zn ratio. The analysis showed no significant differences between the two groups in any of these parameters (Figure 2). However, both groups displayed, on average, a relatively low TE status as compared to healthy adults of the European Prospective Investigation into Cancer and Nutrition (EPIC) study [17], which is used as a reference.

### 2.4. Analysis of Trace Element Status with Regard to C-Reactive Protein

**Figure 3:**
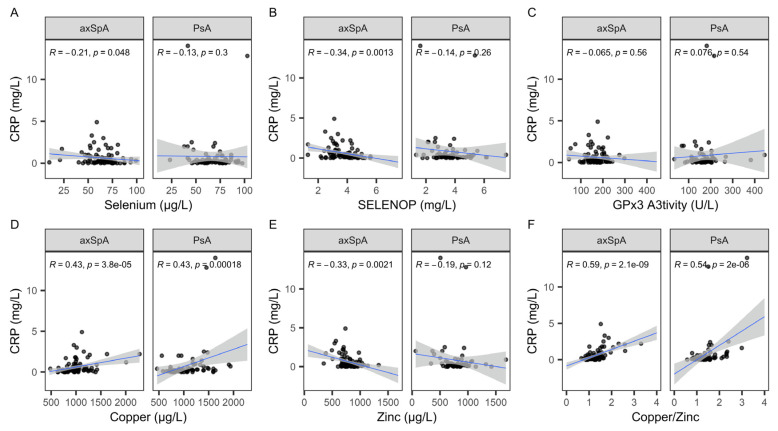
Correlation analysis of the trace element biomarkers with C-reactive protein. The analyses indicated an inverse association of C-reactive protein (CRP) with (**A**) total Se, and (**B**) SELENOP in axSpA, but not in PsA. (**C**) No correlation was found for either group between CRP and GPx3 activity. (**D**) With respect to the other trace elements, there was a positive association in both groups of patients for CRP with serum Cu. (**E**) An inverse association was observed for CRP and serum Zn in axSpA, but not in PsA. (**F**) Both patient groups displayed a highly significant positive association of CRP with the Cu-Zn ratio. Analysis by non-parametric Spearman correlation (R and *p*-values are indicated).

In order to test the hypothesis that TE disturbances are associated with inflammation, all three biomarkers of Se status, along with Cu and Zn and their ratio, were correlated to the prominent inflammation marker C-reactive protein (CRP) (Figure 3). The analysis indicated several significant interactions and some differences between the two diseases. The TE biomarkers Se, SELENOP, and Zn were inversely associated with CRP in axSpA, but not in PsA (Figure 3A,B,E). Serum Cu showed a positive correlation to CRP in both patient groups (Figure 3D), in agreement with the Cu-Zn ratio, which appeared to be the more sensitive parameter for this interaction (Figure 3F).

**Figure 4:**
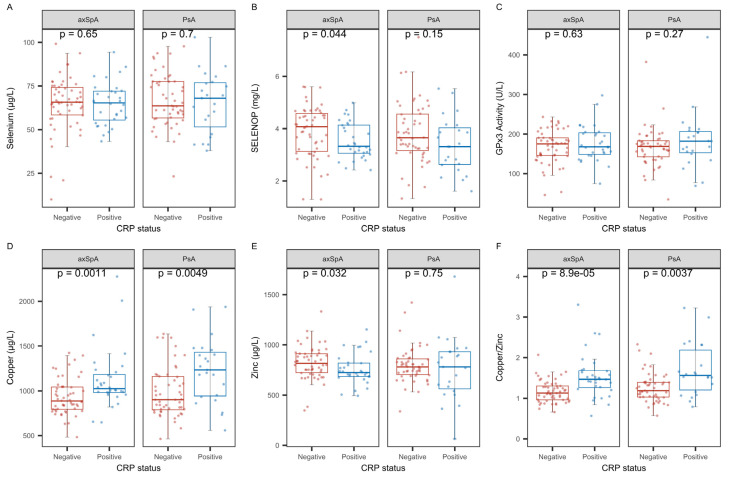
Comparison of TE biomarkers between CRP-positive and CRP-negative patients in both patient groups. The patients were classified according to their CRP status (threshold: 5.0 mg/L). The direct comparison showed no significant differences in (**A**) total serum Se. (**B**) Serum SELENOP was particularly low in CRP-positive axSpA patients. (**C**) There were no differences in serum GPx3 activity in relation to CRP status. (**D**) Total serum Cu was higher in both groups of CRP-positive patients. (**E**) Total Zn was decreased in CRP-positive axSpA patients. (**F**) The serum Cu-Zn ratio was higher in both groups of CRP-positive patients. Analysis by non-parametric group comparisons. *p*-values are indicated.

Next, the interactions between the TE and CRP in the two diseases were analyzed by classifying patients according to their CRP status (Figure 4). The axSpA patients with positive CRP status (CRP > 5.0 mg/L) displayed significantly lower concentrations of SELENOP and Zn (Figure 4B,E) and higher concentrations of Cu and the Cu-Zn ratio (Figure 4D,F), as compared to CRP-negative axSpA patients (CRP < 5.0 mg/L). The CRP-positive PsA patients showed higher serum Cu and a higher Cu-Zn ratio as compared to CRP-negative PsA patients (Figure 4D,F). The parameters total serum Se and GPx activity did not differ between the two groups of iRMD patients (Figure 4A,C).

### 2.5. Associations of TE Status with Regards to Erythrocyte Sedimentation Rate

**Figure 5:**
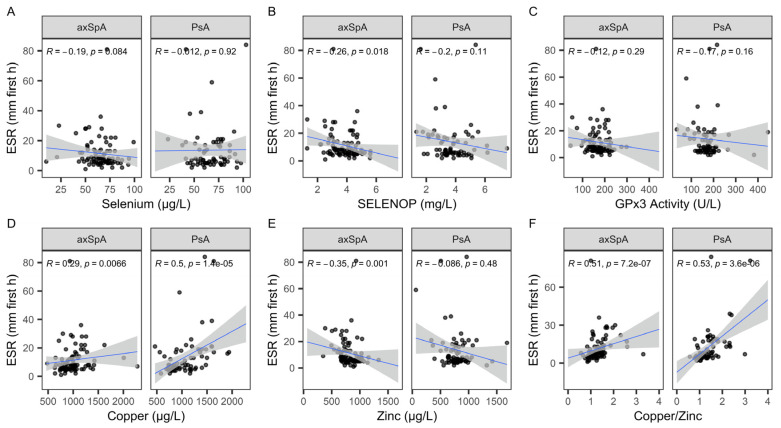
Correlation analysis of the TE biomarkers with ESR in the groups of patients with axSpA and PsA. The patients were classified according to their ESR (threshold: 20 mm/h in the first hour). The analyses indicated no association of CRP with (**A**) total Se, but a significant inverse correlation between (**B**) SELENOP and ESR in axSpA, but not in PsA. (**C**) No correlation was found for either group between ESR and GPx3 activity. (**D**) There was a strong positive association in both groups of patients for serum Cu with ESR. (**E**) An inverse association was observed for ESR and Zn in axSpA, but not in PsA. (**F**) Both patient groups displayed a highly significant positive association of ESR with the Cu-Zn ratio. Analysis by non-parametric Spearman correlation (R and *p*-values are indicated).

Erythrocyte sedimentation rate (ESR) was the second laboratory marker of inflammation in our routine analysis (Figure 5). In general, the analysis accorded with the findings for CRP and indicated an inverse correlation of SELENOP and Zn with ESR in axSpA, but not in PsA (Figure 5B,E), and a general positive correlation of Cu and the Cu-Zn ratio with ESR in both groups of patients (Figure 5D,F).

**Figure 6:**
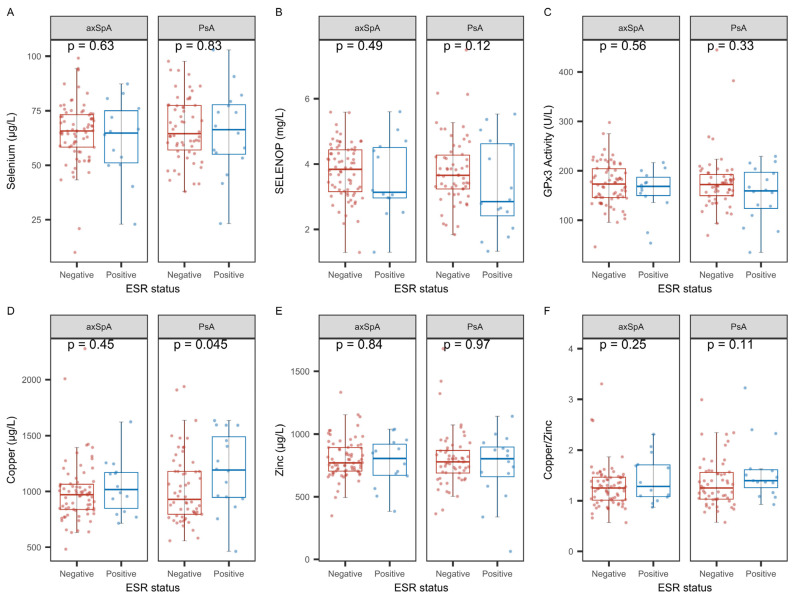
Comparison of TE biomarkers between ESR-positive and ESR-negative patients in both patient groups. The direct comparison showed no significant differences in (**A**) total Se, (**B**) SELENOP, or (**C**) GPx3 activity in relation to ESR (threshold: 20 mm/h in the first hour). (**D**) Total Cu was elevated in ESR-positive PsA patients. No differences were observed in axSpA or PsA patients in relation to ESR status for (**E**) Zn, or (**F**) the Cu- Zn ratio. Analysis by non-parametric group comparisons. *p*-values are indicated.

The interactions between the TE and inflammation were further analyzed by classifying patients according to their ESR status as positive or negative (threshold: 20 mm/h in the first hour). Except for higher Cu in ESR-positive PsA, no differences were observed in relation to ESR status (Figure 6A–F).

### 2.6. Associations of SELENOP Sufficiency with Regard to C-Reactive Protein

**Figure 7:**
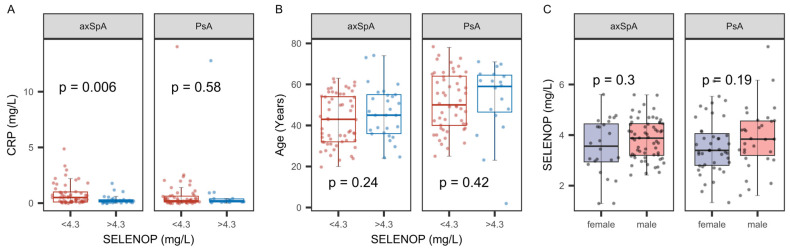
Comparison of CRP status, with respect to SELENOP sufficiency, and potential effects of age and gender. (**A**) The direct comparison indicates a relatively elevated CRP status in axSpA patients with SELENOP deficiency as compared to SELENOP-sufficient axSpA patients. The comparisons of patients indicated no association of (**B**) age or (**C**) gender with the SELENEOP status. Analysis by non-parametric group comparisons. *p*-values are indicated; (threshold (CRP): 5.0 mg/L).

Next, the threshold for SELENOP sufficiency (>4.3 mg/L) was applied to separate patients into well-supplied and SELENOP-deficient, respectively. The comparisons indicated that axSpA patients with SELENOP deficiency had increased CPR as compared to patients with higher SELENOP status (Figure 7A). With respect to confounders, there was no effect of age or gender on SELENOP status (Figure 7B,C).

### 2.7. Analysis of Biomarkers of TE Status with Regard to Remission

**Figure 8:**
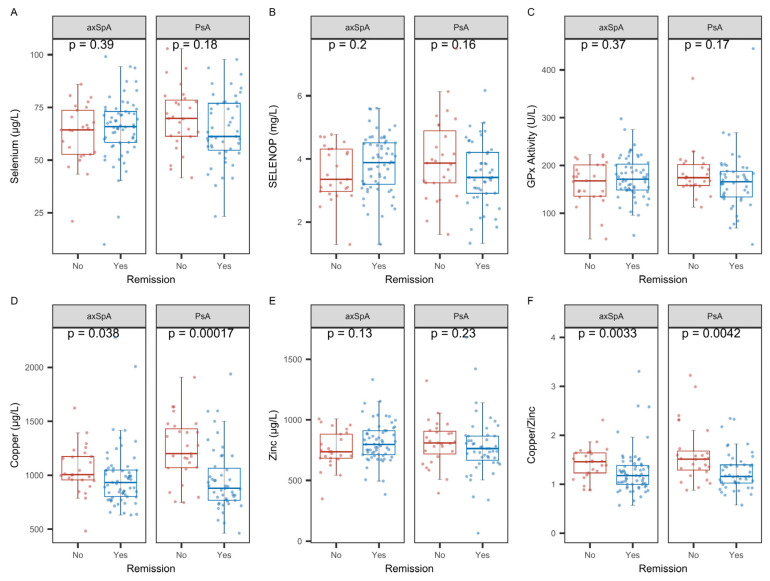
Comparison of biomarkers of trace element status between patients in remission or active disease. The direct comparison did not indicate significant differences in (**A**) total Se, (**B**) SELENOP, or (**C**) GPx3 activity. Differences were observed for (**D**) total Cu, in particular for PsA patients. (**E**) Total Zn was not different between patients in remission or not, whereas (**F**) the ratio of Cu-Zn differed with respect to remission. Analysis by non-parametric group comparisons (R and *p*-values are indicated).

Disease remission in axSpA and PsA is assessed by different scores, e.g., the Bath Ankylosing Spondylitis Disease Activity Index (BASDAI) and the Disease Activity in Psoriatic Arthritis (DAPSA) index. The comparison of TE status with disease remission indicated that among all the parameters tested, serum Cu and the Cu-Zn ratio were the only significantly associated biomarkers, with both being relatively low in remission as compared to ongoing active disease states (Figure 8).

### 2.8. Overview of the Interactions of Trace Elements and Disease Parameters

**Figure 9:**
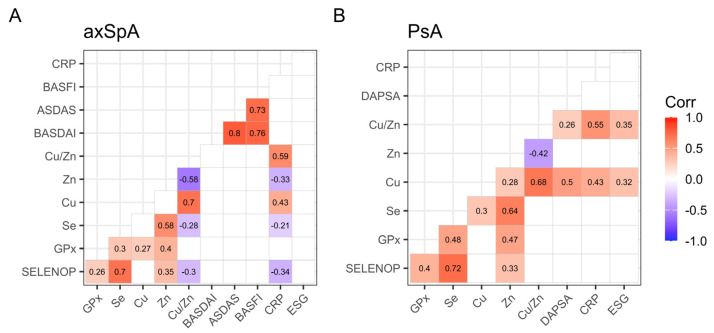
Comparison matrix of trace elements, markers of inflammation, and disease activity scores. (**A**) Spearman’s correlation matrix for axSpA. (**B**) Spearman’s correlation matrix for PsA.

In order to obtain the interaction of the different trace elements with other relevant parameters, we performed correlation analysis with their biomarkers, the indices of inflammation, and standardized, evaluated disease activity and function scores. The results indicated some correlations with systemic inflammatory blood biomarkers, but no correlations with the disease activity scores of axSpA (BASFI, ASDAS, BASDAI) or PsA (DAPSA) were found (Figure 9). A graphical representation of the disease activity scores can be found in the supplementary material (Supplementary Figure S1).

## 3. Discussion

This study provides an overview of biomarkers of TE status in patients with axSpA and PsA, and a comparison with healthy subjects, with a focus on inflammation and remission status. The data indicate a general and consistent TE deficiency in both groups of iRMD patients, without a strong difference between axSpA and PsA. Overall, the results are broadly in line with previous analyses reporting reduced Se and Zn status and a trend towards increased serum Cu concentrations in iRMD [10,13,18,19,20,21]. The verification of serum Cu and the Cu-Zn ratio positively correlating with inflammatory burden and negatively with remission merits consideration for treatment monitoring and patient care. It is noteworthy that despite the positive interaction of inflammation with serum Cu and the Cu-Zn ratio, absolute serum Cu concentrations were below the average of healthy European adults. This finding suggests that hepatic Cu stores apparently failed to mount and support a regular ceruloplasmin and Cu response to inflammation in axSpA and PsA. In how far Cu supplementation would be of clinical benefit under these conditions remains to be determined. The other two important trace elements with high relevance to the immune system, namely Se and Zn, showed the expected inverse correlation with CRP and ESR in axSpA, but not in PsA. This finding was surprising and suggests a different regulation in the two diseases. Also, here, mean SELENOP and Zn levels were below the European average, which could be of pathological relevance, especially in patients with increased systemic inflammation. Since SELENOP expression declines in inflammatory diseases and positively responds to Se intake, it could serve as a suitable biomarker to guide and monitor personalized nutritional and immunosuppressive measures to alleviate disease activity and the inflammatory burden. However, due to the exploratory nature of these analyses, the results were not adjusted for multiple comparisons and need to be interpreted with due caution.

The potential relevance of serum Cu as a biomarker for iRMD reflecting disease activity and positively correlating with ESR and CRP is consistent with previous studies [22,23,24]. Although the data basis is not fully congruent, an increased Cu status was observed in most of the previous reports, which was attributed to its regulation as a positive acute phase reactant in blood, positively associated with disease burden [13,15,24]. At the molecular level, serum Cu is mainly represented by liver-derived ceruloplasmin, and both ceruloplasmin and Cu increase with inflammation in various diseases [15,25,26,27]. In addition to soil levels and thus habitual dietary intake, serum Cu is also affected by endogenous steroid hormones and medications. First-line methotrexate treatment in PsA has been shown to be effective in relieving disease activity and suppressing elevated Cu concentrations [28]. Since a dysregulation of Cu is a consistent finding in iRMD, the question of a causal involvement of Cu in disease risk has been investigated. Mendelian randomization results suggested a positive role of elevated copper status in osteoarthritis, but not in rheumatoid arthritis or ankylosing spondylitis [14,29,30]. Therefore, the positive correlations of the parameters of inflammation with serum Cu and the Cu-Zn ratio in patients with axSpA or PsA are more indicative of a response to disease activity than of a causal relationship. The Cu biomarkers may, therefore, be of potential value in monitoring and assessing the burden of inflammation and treatment efficacy. As both Cu and the Cu-Zn ratio were below the European average in the patients despite their positive acute phase response, supplemental Cu intake merits consideration in order to avoid overt deficiencies and correct Cu status. Respective positive effects of intramuscular Cu administration were observed in previous studies of rheumatoid arthritis [31,32].

Regarding the other essential trace elements, the data indicate low concentrations of the biomarkers of Se status, which is consistent with previous studies [21,32,33,34,35]. Low Se may indicate insufficient habitual dietary intake, which has been described for vitamins and Se in patients with r-axSpA [36]. At the molecular level, serum Se and SELENOP are known to behave as negative acute phase reactants and to inversely correlate with inflammation [9,37,38]. Underlying mechanisms contributing to the inflammation-induced decline of Se and SELENOP in serum include suppression of SELENOP transcription by inflammatory cytokines, along with coordinated downregulation of the various rate-limiting components of hepatic selenoprotein biosynthesis, and a redirection of selenoprotein translation away from SELENOP secretion towards intracellular selenoproteins with antioxidative functions [39,40,41]. Therefore, there may be two potential ways to improve Se status in the patients, namely by supplemental Se supply or by suppressing inflammation. The former has proven to yield some positive effects, and the latter has performed with considerable success in inflammatory arthritis with methotrexate and/or anti-tumor necrosis factor therapy [42,43].

However, when comparing the two patient groups, it was surprising to observe discordant interactions, with the expected inverse association of Se status with CRP and ESR in axSpA, but not in PsA, although both groups had similar levels of inflammation. In psoriasis, it was observed that men and women with a long disease duration had particularly low Se status. The extent to which the autoimmune component contributes to the aberrant relationship between inflammation and Se status in PsA compared to axSpA remains to be analyzed [44].

Serum Zn was relatively low in both patient groups and inversely correlated with CRP and ESR in axSpA, but not in PsA; i.e., it showed a similar pattern to the biomarkers of Se status. Serum Zn was shown to be low in patients with rheumatoid arthritis [15] and PsA [28]. These findings raise the question of disease predisposition or consequence. Mendelian randomization analyses did not show a consistent relationship between genetic predictors of Zn and Se status as causal factors for iRMD [14,29,30]. This is consistent with similar studies examining the potential role of genetic predisposition to Se or Zn deficiency in other joint-involving diseases such as systemic lupus erythematosus or gout [45,46]. However, these analyses are challenging because both serum Se and Zn are regulated mainly by dietary intake. In particular, no limiting protein for serum Zn levels is known, and fluctuations in serum Zn may rather reflect redistribution between different compartments, which is dynamic in nature and strongly affected by dietary intake [47,48]. Likewise, serum Se status is mainly determined by dietary intake and post-transcriptional mechanisms that control the biosynthesis of the circulating transporter SELENOP, which is controlled at the level of translation in the liver [49]. As with Cu, it can be assumed that Se and Zn intake is insufficient in iRMD patients and that their metabolism is impaired by inflammation, leading to the observed relative deficiencies compared to healthy adults. While an analysis of their concentrations provides valuable information on nutritional status and intake, building compound indices, such as, e.g., the Cu-Zn ratio may yield additional information with relation to diagnosis, disease activity, and prognosis, as is shown, e.g., in neonatal infections [50] or for assessing cancer incidence and mortality risks [51,52].

Intervention trials aiming at restoring Cu, Se, or Zn status in iRMD patients by supplementation are scarce and have not shown consistent benefits [32,42,53,54]. However, the study groups were rather small, the dosages and time intervals of supplementation were short, and the observation periods were not particularly long, highlighting a need for sufficiently powered and well-controlled intervention trials to test the potential relevance and efficacy of restoring the immune-relevant TE to reference levels.

Another interesting finding of our study is the correlation of the results for trace elements with systemic blood markers of inflammation, such as CRP and ESR. It is remarkable that, despite the fact that these markers are known to be positive in only about 40–50% of patients with axSpA [55], the correlations found to trace element results were at least moderate. This finding confirms the reliability of measuring trace elements in patients with SpA and particularly with axSpA, with this lack of objective markers of systemic inflammation. In addition, this finding is particularly interesting in the background of the lack of correlations that we found to the clinical scores of disease activity or function, which are known to be susceptible to subjective interferences with comorbidities associated with disease chronicity, such as generalized pain and stiffness.

The strengths of this study include the well-characterized and balanced study groups of patients with axSpA and PsA, as well as a comprehensive analysis of their TE serum levels and additional biomarkers. However, our study is not without limitations. The data were only obtained from a single sample per patient; the study design is mainly descriptive and observational and, therefore, does not allow for causal conclusions. Furthermore, the results, interpretations, and conclusions are limited as the study groups were recruited from a Central European population; therefore, the results cannot be extrapolated to other populations with a different genetic background and dissimilar habitual TE intake.

We conclude that the studied groups of patients with axSpA and PsA had a general TE deficiency, with low concentrations of the biomarkers of Se status and a deficiency of Cu and Zn in serum. These deficits are likely secondary to poor nutritional supply, disease-related dysregulation of their metabolism, or higher demands that are not regularly met by the normal diet. Cu concentrations and the Cu-Zn ratio positively reflected inflammation and were associated with non-remission. Inflammation was inversely related to SELENOP status in axSpA, highlighting a disease-relevant deficiency that is amenable to nutritive correction, ideally in an individualized manner, as needed. In view of the low Se status, deficient Se transport and suppressed selenoprotein biosynthesis have been causally related to oxidative damage, disturbed metabolic regulation, lipid peroxidation, and induction of cell death by ferroptosis [11,42,56,57]. It appears meaningful to avoid Se and SELENOP deficiency by improved dietary supply or personalized supplementation efforts. The chances of positively affecting disease course and reducing parameters of joint inflammation and damage are relatively high, as exemplified by successful supplementation trials in Kashin-Beck osteoarthropathy in the endemic regions low in Se status [58,59,60]. As surplus Se intake has also been associated with adverse effects, a prudent and personalized strategy with longitudinal monitoring of such interventions is warranted to avoid side effects and toxicity [61,62].

## 4. Materials and Methods

### 4.1. Study Population

The present study was initiated at the university hospital and tertiary rheumatology center Rheumazentrum Ruhrgebiet (ethics approval: Votum Reg. Nr. 15-5309, Ethik-Kommission der Medizinischen Fakultät, Ruhr-Universität Bochum) with biomaterial from the prospective LORE cohort (Long-Term Observation of Patients Diagnosed with a Chronic Inflammatory Disease from the Rheumatic Form Group) established at the same center (ethics approval: 20-6939, Ethik-Kommission der Medizinischen Fakultät, Ruhr-Universität Bochum). The LORE cohort consists of patients with a clinical diagnosis of chronic inflammatory rheumatic disease, recruited from a prospective daily practice observational study. As part of the registry project, patient characteristics, disease status, disease activity, structural changes in imaging, possible organ damage, functional capacity, comorbidities, and social participation, as well as the therapy carried out (medicinal and non-medicinal), are recorded. The analyses included patients with a clinical diagnosis of axSpA or PsA with complete data on disease activity, functional capacity, comorbidities, pain levels (numerical pain rating scale (NRS pain) [63], Bath Ankylosing Spondylitis Disease Activity Index (BASDAI) [64], Ankylosing Spondylitis Disease Activity Score (ASDAS-CRP) [2], and Disease Activity score for PsA (DAPSA) [65], where other reasons for having high inflammatory disease activity markers were excluded. Given the continuum of symptoms between nr-axSpA and r-axSpA, with the former usually progressing to the more severe form r-axSpA over time, no distinction was made between these two forms of the disease in this explorative study.

### 4.2. Trace Element Status Assessment

Serum samples were collected from the biomaterial bank at Ruhr-Universität Bochum, transferred on dry ice to the analytical lab at Charité-Universitätsmedizin Berlin, and analyzed. Three relevant TE (Cu, Se, and Zn) were quantified in serum by total reflection X-ray fluorescence (TXRF) analysis, as described [66]. To this end, serum was diluted with a gallium standard, applied to clean glass disks, and dried. A benchtop TXRF spectrometer (T-STAR, Bruker Nano GmbH, Berlin, Germany) was used for X-ray irradiation and recording of the fluorescence emitted by the sample. The analysis of the resulting fluorescence spectrum by extracting the areas under the emission peaks at particular wavelengths yielded the TE-specific concentrations. Besides total TE, two complementary protein biomarkers of Se status were determined, namely the enzymatic activity of GPx3 and the concentration of the Se transporter SELENOP. Briefly, GPx3 activity was measured by a coupled enzyme activity assay with glutathione and tert-butyl-hydroperoxide as substrates, as described [67]. The reduction of glutathione catalyzed by glutathione reductase led to a decrease in NADPH, which was quantified at 340 nm as an indicator of enzymatic activity. To ensure consistency in reaction conditions, a standard serum was incorporated into all measurement runs, yielding a coefficient of variation (CV) below 15% throughout the analyses. SELENOP levels were quantified using a chemiluminescent sandwich immunoassay performed on an automated laboratory system (iSYS, Immunodiagnostic Systems Holdings Ltd. (ids), Frankfurt, Germany), using monoclonal antibodies, standards, and controls from a validated commercial assay (selenOtest, selenOmed GmbH, Berlin, Germany), as described [68]. Both the inter- and intra-assay CVs were below 5% during the analyses, as determined with a constant control serum.

### 4.3. Statistical Analyses

Statistical analyses were conducted using the R Studio Integrated Development Environment, version 4.1.1, implementing the packages “tidyverse”, “ggplot2”, and “gtsummary”. For all continuous variables, normality was evaluated using the Shapiro-Wilk test. Continuous variables are expressed as median with interquartile range (IQR). Comparisons between two groups were made using the Wilcoxon rank-sum test. The correlation between biomarkers was assessed using Spearman’s rank correlation test. All analyses were two-sided, and *p*-values less than 0.05 were considered statistically significant (* *p* < 0.05, ** *p* < 0.01, *** *p* < 0.001, and **** *p* < 0.0001).

## Figures and Tables

**Table 1:** Characteristics of the patients with ankylosing spondylitis (axSpA) and psoriatic arthritis (PsA).

Characteristic	axSpA n = 84	PsA n = 76
**Age**	43.0 (21.3)	52.5 (23.0)
(Missing)	0	2
**Sex**		
Female	24 (29%)	44 (58%)
**Weight**	85.0 (21.8)	89.0 (28.0)
(Missing)	48	53
**CRP Status**		
Positive, >0.5 mg/dL	32 (38%)	23 (30%)
**ESR Status**		
Positive, >20 mm/h	14 (17%)	16 (21%)
1Median (IQR); n (%)		

## Data Availability

The data presented in this study are available on request from the corresponding author.

## Supplementary Materials

The following supporting information can be downloaded at: https://www.mdpi.com/article/10.3390/ijms26104924/s1.

## Abbreviations

The following abbreviations are used in this manuscript:

Se	selenium
Cu	copper
Zn	zinc
TE	trace element(s)
axSpA	axial spondyloarthritis
PsA	psoriatic arthritis
TXRF	total reflection X-ray fluorescence
SELENOP	selenoprotein P
GPx3	extracellular glutathione peroxidase
CRP	C-reactive protein
ESR	erythrocyte sedimentation rate
SIJ	sacroiliac joints
r-axSpA	classic ankylosing spondylitis
nr-axSpA	non-radiographic ankylosing spondylitis
iRMD	rheumatic musculoskeletal disease(s)
GPx1	cellular glutathione peroxidase
GPx4	phospholipid-hydroperoxide glutathione peroxidase
EPIC	European Prospective Investigation into Cancer and Nutrition
BASDAI	Bath Ankylosing Spondylitis Index
DAPSA	Disease Activity in Psoriatic Arthritis
LORE cohort	Long-Term Observation of Patients Diagnosed with a Chronic Inflammatory Disease from the Rheumatic Form Group cohort
