# Supplementary material for: Trace Element Deficiency in Axial Spondyloarthritis and Psoriatic Arthritis in Relation to Markers of Inflammation and Remission
Source: Int J Mol Sci. 2025 May 21;26(10):4924. doi: 10.3390/ijms26104924 (PMC12112114; doi:10.3390/ijms26104924)
Supplement: Supplementary file 1 [file ijms-26-04924-s001.zip › ijms-3544634-supplementary.pdf]

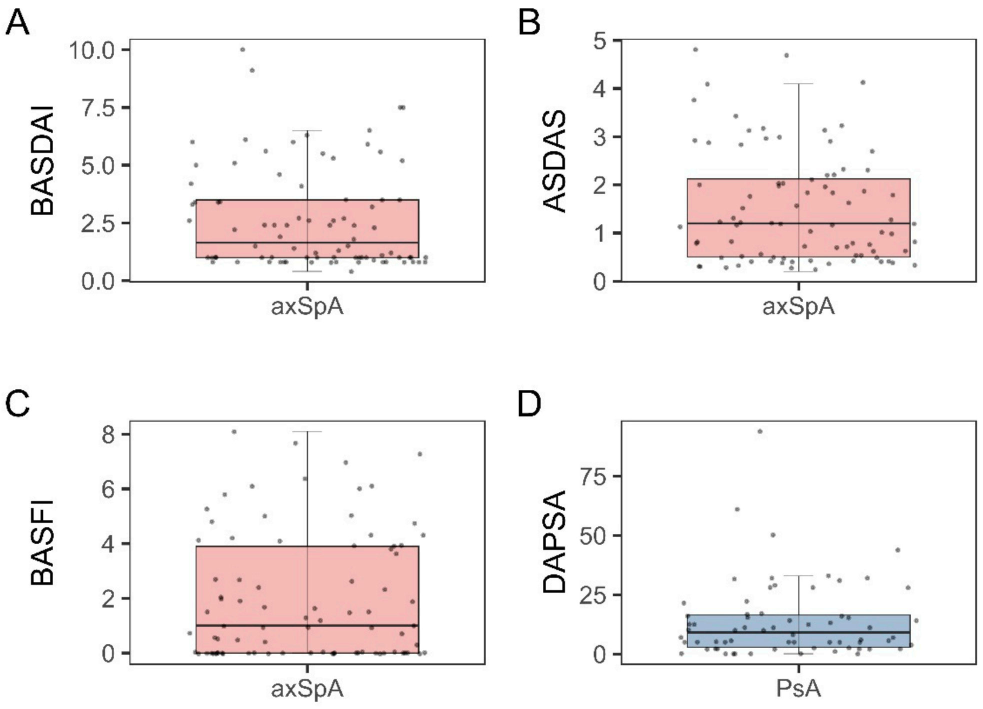

Supplementary Figure S1. Overview on disease symptom scores of the two groups of patients with axial spondyloarthritis (axSpA) and psoriatic arthritis (PsA), respectively. The clinical diagnosis included characterization of patients by disease-specific compound scoring systems; pain levels (numerical pain rating scale (NRS pain), Bath Ankylosing Spondylitis Disease Activity Index (BASDAI), Ankylosing Spondylitis Disease Activity Score (ASDAS-CRP), and Disease Activity score for PsA (DAPSA). The individual results along with box plots and whiskers are displayed.
